# Supplementary material for: Macrocyclization and Backbone Rearrangement During RiPP Biosynthesis by a SAM-Dependent Domain-of-Unknown-Function 692
Source: ACS Cent Sci. 2023 Apr 24;9(5):1008–18. doi: 10.1021/acscentsci.3c00160 (PMC10214503; doi:10.1021/acscentsci.3c00160)
Supplement: Supplementary file 4 — oc3c00160_si_004.pdf [file oc3c00160_si_004.pdf]

oc-2023-00160c.R1

Name: Peer Review Information for "Macrocyclization and backbone rearrangement during RiPP biosynthesis by a SAM-dependent domain-of-unknown-function 692"

First Round of Reviewer Comments

Reviewer: 1

Comments to the Author

This article focuses on a remarkable peptide macrocyclization and backbone rearrangement in a RiPP natural product encoded in the genome of Chryseobacterium. Enzymes belonging to the DUF692 family have previously been found in two different RiPP contexts: methanobactin biosynthesis and in the biosynthesis of thiaglutamate. This work constructed a sequence similarity network for DUF692 enzymes and found a large cluster in Chryseobacterium. Heterologous expression of two of the genes from these BGCs, ChrH and ChrI, with the gene for the precursor ChrA, led to a modified ChrA product as judged by mass spectrometry. The rest of the paper uses a combination of mass spectrometry, NMR, and isotope labeling experiments to deduce the structure of the modification. Both heterologous expression and in vitro approaches were employed. Key findings include 1) that ChrH and ChrI function together, 2) that ChrH binds multiple iron ions, 3) SAM is used a methyl donor, generating a thiomethyl group on cysteine. ChrHI function to install both a thioether linked macrocycle and an imidazolidinedione moiety. To arrive at this final structure, a dizzying array of bond movements must occur, though there is some precedent for this in the DUF692 enzymes. The excision of the Cys8 beta carbon, for example, resembles the chemistry that occurs in thiaglutamate biosynthesis. Overall, the novelty of this new chemical moiety, the thorough NMR and mass spec analysis, and the technical achievement of reconstituting a new RiPP pathway in vitro (with a membrane protein to boot) make this work worthy of publication in my opinion. I have several questions and suggestions given below.

1) In figure 1, the meaning of n=115 in part b is not clear. Should this have been placed in the SSN itself to indicate that there are 115 sequences in the ChrH node? Not to be too picky, but Figure 1C is not technically a multiple sequence alignment, it is a sequence logo. I would also suggest the authors include the accession numbers of the sequences used to generate the sequence logo in the supporting information.

2) Page 6, line 18. When I first read this passage I initially thought that only Cys8 was being replaced with the C13-labeled amino acid. It becomes clear in the next few sentences that this is not the case, but I might suggest saying that the protein was prepared by heterologous expression

3) In the NMR sections, I could not find the concentration of the samples analyzed. Also, the authors employed both water and DMSO as NMR solvents. While it is clear in the SI which spectra were collected in which solvents, some rationale for the switch between solvents could be useful for the reader.

4) In the alphafold model for ChrH, the authors do not discuss any putative SAM binding sites, which are usually relatively easy to identify within proteins.  Do the authors think SAM binds to ChrH or to ChrI? ChrH seems unlikely to bind SAM given that MbnB does not.  Suggest possibly adding an alphafold model of ChrI to the SI as well.

5) The instability of the -36 Da putative intermediate was surprising, especially given some of the proposed structures for this intermediate in the SI.  Could the authors give some details about the “rapid degradation under purification conditions” mentioned on page 10 of the manuscript?

6) Also on page 10, line 8, I believe Figure 3B should be Figure 5B

7) While the NMR figures in the main text use arrows to point to specific correlations, I found some of the NMR figures in the SI hard to follow.  One example of this is Figure S6F where it is unclear which peak the label is pointing at.  Figure S7B is another one where arrows pointing to the peak of interest would help readability.

8) In the version I received for review, there is a typo in the first row of Table S2 (missing Greek letter)

Reviewer: 2

Comments to the Author

Enzymes in the DUF692 family have recently emerged as biocatalysts that carry out intriguing transformations in RiPP biosynthetic pathways. In this manuscript, Ayikpoe et al. add a new, and arguably the most unusual, reaction to the repertoire. The enzyme ChrH is found to catalyze a remarkable multi-step modification involving numerous bond-breaking/bond-forming steps that ultimately result in an imidazolidinedione and a thioether macrocycle. The structural work is in general solid and the paper methodical and well-written. While several aspects remain to be investigated - e.g. mechanism, structure, etc - the manuscript provides a nice starting point for these future efforts. I only have minor suggestions to further clarify and possibly enhance the manuscript:

- page 1, line 41: I would remove 'extensively' as some mature RiPPs are synthesized with only minimal modifications.

- page 2, line 22: Please reconsider naming the product of the pathway as the ending 'bactin' is often used for siderophores.

- Figure 3: It is difficult to discern the product of the ChrH reaction with the correlation arrows in panel F. I would suggest that the authors add another panel with the 'clean' structure of the enzymatic product.

- Figure 5A: please replace 'HI' with 'ChrHI' as in Figures 5B and 2.

- I wonder if it makes sense to highlight the imidazolidinedione further in the discussion: the previously examined DUF692 enzymes modify Cys residues and so the thioether macrocycle, while still novel, is consistent with what is known about these enzymes so far. The imidazolidinedione, however, is quite unexpected.

- page 11, line 8: replace 'residue' with 'resides'

Reviewer: 3

Comments to the Author

This manuscript by van der Donk and coworkers describes studies on the reaction catalyzed by a RiPP peptide maturase belonging to DUF 692. Previous studies with homologs MbnB and TglH have both indicated unprecedented reactivity involving Cys containing peptides. The homolog from Chryseobacterium studied here is not an exception. It appears to catalyze the conversion of a Cys to an imidazolidinedione heterocyclic. The authors show that incubation of the ChrA peptide with ChrH and ChrI (in vitro or in vivo) both lead to the same product. The enzyme appears to bind 2 or more iron ions (as with the MbnB and TglH) and oxygen is presumable required to activate the substrate by C-H transfer. Remarkably, the reaction utilizes SAM as a methyl donor and the source of the methyl group appending the final structure. The authors provide several additional possible mechanistic proposals in the SI. As best as I can tell, the product is well characterized by NMR and HR-MS/MS experiments. Overall, this is a highly intriguing reaction in biogenesis of RiPP natural products.

Author's Response to Peer Review Comments:

*Our responses are in blue italics font*

Reviewer: 1

Recommendation: Publish in ACS Central Science after minor revisions noted.

Comments:
This article focuses on a remarkable peptide macrocyclization and backbone rearrangement in a RiPP natural product encoded in the genome of Chryseobacterium. Enzymes belonging to the DUF692 family have previously been found in two different RiPP contexts: methanobactin biosynthesis and in the biosynthesis of thiaglutamate. This work constructed a sequence similarity network for DUF692 enzymes and found a large cluster in Chryseobacterium. Heterologous expression of two of the genes from these BGCs, ChrH and ChrI, with the gene for the precursor ChrA, led to a modified ChrA product as judged by mass spectrometry. The rest of the paper uses a combination of mass spectrometry, NMR, and isotope labeling experiments to deduce the structure of the modification. Both heterologous expression and in vitro approaches were employed. Key findings include 1) that ChrH and ChrI function together, 2) that ChrH binds multiple iron ions, 3) SAM is used a methyl donor, generating a thiomethyl group on cysteine. ChrHI function to install both a thioether linked macrocycle and an imidazolidinedione moiety. To arrive at this final structure, a dizzying array of bond movements must occur, though there is some precedent for this in the DUF692 enzymes. The excision of the Cys8 beta carbon, for example, resembles the chemistry that occurs in thiaglutamate biosynthesis. Overall, the novelty of this new chemical moiety, the thorough NMR and mass spec analysis, and the technical achievement of reconstituting a new RiPP pathway in vitro (with a membrane protein to boot) make this work worthy of publication in my opinion. I have several questions and suggestions given below.

1) In figure 1, the meaning of n=115 in part b is not clear. Should this have been placed in the SSN itself to indicate that there are 115 sequences in the ChrH node? Not to be too picky, but Figure 1C is not technically a multiple sequence alignment, it is a sequence logo. I would also suggest the authors include the accession numbers of the sequences used to generate the sequence logo in the supporting information.

*Response: We thank the reviewer for the comment. The "n=115" represents the number of sequences used to generate the sequence logo and we replaced "multiple sequence alignment" with "sequence logo" in the legend for Figure 1C. The sequence of the precursor peptides used to generate the sequence logo has been included in the SI as an attachment file (Table S6). We also submitted the cytoscape file for Fig. 1B.*

2) Page 6, line 18. When I first read this passage I initially thought that only Cys8 was being replaced with the C13-labeled amino acid. It becomes clear in the next few sentences that this is not the case, but I might suggest saying that the protein was prepared by heterologous expression

*Response: We agree that this was not well worded and now explicitly state that the labeling is on both Cys5 and Cys8. We also added "by heterologous expression in E. coli" to the sentence on page 6, line 18 to indicate that ChrA selectively labeled at Cys8 and Cys5 was prepared in E. coli.*

3) In the NMR sections, I could not find the concentration of the samples analyzed. Also, the authors employed both water and DMSO as NMR solvents. While it is clear in the SI which spectra were collected in which solvents, some rationale for the switch between solvents could be useful for the reader.

*Response: We have added a few sentences describing the concentrations of NMR samples and the rationale for solvent selection to the SI under the NMR acquisition section. i.e., "The concentrations of ChrA*, $^{13}$C-beta-Cys ChrA*, $^{13}$C-carbonyl-Cys ChrA*, and $^{15}$N-ChrA* in DMSO-d6 were estimated to be 1.0 mM, 0.9 mM, 0.4 mM, and 0.5 mM, respectively, and ChrA in 90% $H_2O$+10% $D_2O$ was estimated to be 0.5 mM. These concentrations were estimated by the qNMR method. The DMSO-d6 solvent was chosen for ChrA* for most of the experiments because it gave the best solubility and sharper linewidth. ChrA dissolved in 90%$H_2O$+10%$D_2O$ also gave good solubility and linewidth and gave better resolution for the alpha-protons of Gly9."*

4) In the alphafold model for ChrH, the authors do not discuss any putative SAM binding sites, which are usually relatively easy to identify within proteins. Do the authors think SAM binds to ChrH or to ChrI? ChrH seems unlikely to bind SAM given that MbnB does not. Suggest possibly adding an alphafold model of ChrI to the SI as well.

*Response: We thank the reviewer for raising this point. The truth is that we do not know which protein catalyzes the methylation reaction and we now specifically mention this. We think that SAM binds to ChrH and not ChrI but at present we do not have specific support for that statement. Alphafold multimer was used to predict the structure of ChrI in complex with ChrH and ChrA and we now include this predicted structure in the SI (Figure S15). ChrI is predicted to be an integral membrane protein with three transmembrane helices with a small stretch of residues interacting with both ChrH and ChrA. The canonical Rossmann fold for binding SAM was not present in the predicted structures of either ChrH or ChrI. ChrI is ~80-100 residues larger than MbnB and it is possible that this difference allows the formation of a non-canonical SAM binding site. AlphaFold has a long unstructured loop that is not seen in MbnB. We have altered the text in some places to avoid the impression that we know that ChrH catalyzes the methylation event. Future studies will focus on obtaining structures of ChrH in complex with ChrA and ChrI as well as SAM to probe their interactions.*

5) The instability of the -36 Da putative intermediate was surprising, especially given some of the proposed structures for this intermediate in the SI. Could the authors give some details about the "rapid degradation under purification conditions" mentioned on page 10 of the manuscript?

*Response: We acknowledge the significance of characterizing the -36 Da putative intermediate. The purification of the peptide requires acidic conditions, however, and the -36 Da species seemingly degrades under those acidic conditions as we do not recover it. Future studies will focus on trying to find conditions that allow investigating the structure of the shunt product/intermediate.*

6) Also on page 10, line 8, I believe Figure 3B should be Figure 5B

*Response: Correct! Figure 3B has been changed to Figure 5B. We very much appreciate the careful read!*

7) While the NMR figures in the main text use arrows to point to specific correlations, I found some of the NMR figures in the SI hard to follow. One example of this is Figure S6F where it is unclear which peak the label is pointing at. Figure S7B is another one where arrows pointing to the peak of interest would help readability.

*Response: We thank the reviewer for the suggestions. Arrows have been included in all the NMR spectra in Fig. S6 and S7 to point to significant peaks used to assign the structure of ChrA*.*

8) In the version I received for review, there is a typo in the first row of Table S2 (missing Greek letter)

*Response: Thanks! The typo in Table S2 has been corrected.*

Additional Questions:
Quality of experimental data, technical rigor: High

Significance to chemistry researchers in this and related fields: High

Broad interest to other researchers: High

Novelty: Top 5%

Is this research study suitable for media coverage or a First Reactions (a News & Views piece in the journal)?: No

Reviewer: 2

Recommendation: Publish in ACS Central Science after minor revisions noted.

Comments:
Enzymes in the DUF692 family have recently emerged as biocatalysts that carry out intriguing transformations in RiPP biosynthetic pathways. In this manuscript, Ayikpoe et al. add a new, and arguably the most unusual, reaction to the repertoire. The enzyme ChrH is found to catalyze a remarkable multi-step modification involving numerous bond-breaking/bond-forming steps that ultimately result in an imidazolidinedione and a thioether macrocycle. The structural work is in general solid and the paper methodical and well-written. While several aspects remain to be investigated - e.g. mechanism, structure, etc - the manuscript provides a nice starting point for these future efforts. I only have minor suggestions to further clarify and possibly enhance the manuscript:

- page 1, line 41: I would remove 'extensively' as some mature RiPPs are synthesized with only minimal modifications.

*Response: We agree. The word extensively has been removed in line 41, page 1.*

- page 2, line 22: Please reconsider naming the product of the pathway as the ending 'bactin' is often used for siderophores.

*Response: We looked into the prior use of the suffix bactin. While cyanobactin is a term for a very large RiPP class that does not have any proven siderophore function, we did indeed find that enterobactin and mycobactin are siderophores. To prevent any suggestion that we know the function of the final product, we have renamed the final product as chryseobasin. We thank the reviewer for pointing out the use of -bactin as suffix for siderophores.*

- Figure 3: It is difficult to discern the product of the ChrH reaction with the correlation arrows in panel F. I would suggest that the authors add another panel with the 'clean' structure of the enzymatic product.

*Response: A new panel was added to Figure 3 to show the proposed 'clean' structure of ChrA*.*

- Figure 5A: please replace 'HI' with 'ChrHI' as in Figures 5B and 2.

*Response: Agreed. HI has been replaced with ChrHI.*

- I wonder if it makes sense to highlight the imidazolidinedione further in the discussion: the previously examined DUF692 enzymes modify Cys residues and so the thioether macrocycle, while still novel, is consistent with what is known about these enzymes so far. The imidazolidinedione, however, is quite unexpected.

*Response: We agree that the imidazolidinedione is unexpected but we do think that its formation is already highlighted as we mentioned its formation in the Abstract, in the caption of Figure3, in the discussion of the mechanism, and in the conclusion. We believe that the formation of the*

*imidazolidinedione is directly coupled to the formation of the two thioaminals and hence we prefer to discuss them all together.*

- page 11, line 8: replace 'residue' with 'resides'

*Response: Thank you! Residue has been changed to resides*

Additional Questions:
Quality of experimental data, technical rigor: Top 1%

Significance to chemistry researchers in this and related fields: Top 1%

Broad interest to other researchers: Top 5%

Novelty: Top 1%

Is this research study suitable for media coverage or a First Reactions (a News & Views piece in the journal)?: No

Reviewer: 3

Recommendation: Publish in ACS Central Science without change.

Comments:
This manuscript by van der Donk and coworkers describes studies on the reaction catalyzed by a RiPP peptide maturase belonging to DUF 692. Previous studies with homologs MbnB and TglH have both indicated unprecedented reactivity involving Cys containing peptides. The homolog from Chryseobacterium studied here is not an exception. It appears to catalyze the conversion of a Cys to an imidazolidinedione heterocyclic. The authors show that incubation of the ChrA peptide with ChrH and ChrI (in vitro or in vivo) both lead to the same product. The enzyme appears to bind 2 or more iron ions (as with the MbnB and TglH) and oxygen is presumable required to activate the substrate by C-H transfer. Remarkably, the reaction utilizes SAM as a methyl donor and the source of the methyl group appending the final structure. The authors provide several additional possible mechanistic proposals in the SI. As best as I can tell, the product is well characterized by NMR and HR-MS/MS experiments. Overall, this is a highly intriguing reaction in biogenesis of RiPP natural products.

*We thank the reviewer for the supportive comments.*

Quality of experimental data, technical rigor: Top 1%

Significance to chemistry researchers in this and related fields: Top 1%

Broad interest to other researchers: Top 1%

Novelty: Top 1%

Is this research study suitable for media coverage or a First Reactions (a News & Views piece in the journal)?: No

oc-2023-00160c.R2

Name: Peer Review Information for "Macrocyclization and backbone rearrangement during RiPP biosynthesis by a SAM-dependent domain-of-unknown-function 692"

Second Round of Reviewer Comments

Reviewer: 1

Comments to the Author

The authors have satisfactorily addressed my concerns. The only remaining issue from my perspective is that the page numbers have some error in the supporting information document.

Author's Response to Peer Review Comments:

AU EMAIL: Please label as "email"

RESPONSE: DONE

SI PARAGRAPH: If the manuscript is accompanied by any supporting information for publication, a brief description of the supplementary material is required in the manuscript. The appropriate format is: Supporting Information. Brief statement in non-sentence format listing the contents of the material supplied as Supporting Information.

RESPONSE: DONE

GENERAL REF FORMATTING: Periodical references should contain authors' surnames followed by initials, article title, journal abbreviation, year, volume number, and page range. Refs with more than 10 authors should list the first 10 and then be followed by "et al."

RESPONSE: WE added page range and listed the first 10 followed by et al for papers with >10 authors

Web sources must include access date.

RESPONSE: we have no web sources

TOC MISSING: Provide a TOC image per journal guidelines (3.25 in. × 1.75 in. (8.25 cm × 4.45 cm) ; on the last page of the Manuscript) with the heading "TOC Graphic" above the graphic. Make sure to designate the file as "Graphic for Manuscript."

RESPONSE: WE had a TOC graphic as separate graphic but have now also added it on the last page of the manuscript.

SYNOPSIS MISSING: The synopsis should be no more than 200 characters (including spaces) and should reasonably correlate with the TOC graphic. The synopsis is intended to explain the importance of the

article to a broader readership across the sciences. Please place your synopsis in the manuscript file after the TOC graphic.

RESPONSE: we added synopsis

SI PG#S: The supporting information pages must be numbered consecutively, starting with page S1.

RESPONSE: we had supporting info pages numbered and starting with S1 but the table of contents was not correct which is likely what the reviewer meant. We have now corrected that.
